# Temporal Profile of the Microbial Community and Volatile Compounds in the Third-Round Fermentation of Sauce-Flavor *baijiu* in the Beijing Region

**DOI:** 10.3390/foods13050670

**Published:** 2024-02-22

**Authors:** Weiwei Li, Hui Zhang, Runnan Wang, Chengnan Zhang, Xiuting Li

**Affiliations:** 1 Beijing Advanced Innovation Center for Food Nutrition and Human Health, Beijing Technology and Business University (BTBU), Beijing 100048, China; liweiwei.0304@163.com (W.L.); 18602699565@163.com (H.Z.); w905308623@163.com (R.W.); 2 Beijing Engineering and Technology Research Center of Food Additives, Beijing Technology and Business University (BTBU), Beijing 100048, China; 3 Department of Exercise Biochemistry, Exercise Science School, Beijing Sport University, Beijing 100084, China; zhangcn@btbu.edu.cn

**Keywords:** sauce-flavor *baijiu*, microbial community succession, changes in flavor compounds, regional flavor characteristics

## Abstract

Sauce-flavor *baijiu* produced in the Beijing and Guizhou regions has regional characteristic flavors, but the differences in flavor compounds and reasons for their formation remain unclear. The sauce-flavor *baijiu* brewing process involves several rounds of fermentation. In this study, we investigated the temporal distribution of microbial communities and flavor substances during the third round of sauce-flavor *baijiu* fermentation in the Beijing region, and we then compared and analyzed the differences of flavor substances and microorganisms in the fermented grains of sauce-flavor *baijiu* in the Beijing and Guizhou regions. It was found that 10 bacterial genera and 10 fungal genera were dominant in the fermented grains. The acidity of the fermented grains had a significant driving effect on the microbial community succession. A total of 81 volatile compounds were identified and quantified in the fermented grains, of which esters and alcohols were relatively abundant. The differences in 30 microbial community compositions and their resulting differences in terms of the fermentation parameters of fermented grains are responsible for the differences in the profiles of flavor compounds between sauce-flavor *baijiu* produced in the Beijing and Guizhou regions.

## 1. Introduction

*Baijiu* has a history of thousands of years and plays an important role in traditional Chinese culture and people’s daily life [1,2,3]. The fermentation process of *baijiu* is generally carried out with single or mixed grains including rice [4], wheat, maize, and sorghum in an open or semi-open fermentation environment [5]. *Baijiu*’s unique fermentation technique, ecological setting, and microecological structure all have a direct impact on its quality [6,7]. According to the different flavors of *baijiu*, it can be divided into 12 flavor types. Among them, strong-flavor *baijiu*, sauce-flavor *baijiu*, and light-flavor *baijiu* are generally considered as the three basic types of *baijiu* [8]. The sauce-flavor *baijiu* is very popular with the public because of its unique flavor [9,10].

For sauce-flavor *baijiu*, the main flavor components have not yet been cracked. The unique flavor of sauce-flavor *baijiu* mainly depends on brewing technology and microbial metabolism [11,12]. The brewing process of sauce-flavor *baijiu* takes approximately one year and seven rounds (Figure S3). The fermentation process of each round includes heap fermentation and pit fermentation. In the first round, sorghum and *daqu* are used as the raw material and fermented starter, respectively (Figure S3). Fresh sorghum is ground into powder, steamed, and cooled to room temperature. The sorghum powder is then mixed with *daqu* for heap fermentation. After 3–5 days, it is moved into the pit, covered with rice husks and pit mud, and fermented for 30 days (Figure S3). At the end of pit fermentation, the fermented grains are remixed with sorghum and *daqu* powder for further heap and pit fermentation. After two rounds of heap and pit fermentation, the fermented grains are distilled to obtain the first-round *baijiu* [13]. In the second round, the distilled fermented grains from the first round are firstly mixed with the *daqu* powder and then used for heap fermentation and pit fermentation. At the end of the second round, the second-round *baijiu* is distilled. The manufacture processes of the third to seventh rounds are the same as that of the second round. The first- to seventh-round *baijiu* obtained at the end of the brewing process of sauce-flavor *baijiu* are stored for 3 years and blended into the product [14]. The flavor characteristics of seven-round *baijiu* differ significantly. The first- and second-round *baijiu* are astringent; the third-, fourth-, and fifth-round *baijiu* are of better quality; and the sixth- and seventh-round *baijiu* become worse, with a bitter and burnt taste [15]. In general, the third-, fourth-, and fifth-round *baijiu* are used in larger quantities in the blending process of sauce-flavor *baijiu* production, which results in their important role in determining the flavor and quality of product.

Interestingly, there are obvious sensory differences in the flavor of sauce-flavor *baijiu* in different regions; one of the reasons is the difference in the structure of microbial communities during the brewing process [15,16,17]. The main regions in China that produce sauce-flavor *baijiu* include the Moutai region, Xishui region, Guizhou region, Sichuan region, Beijing region, and Tianjin region [9,13,15,18]. The microbial fermentation process is the process of making *baijiu*’s flavor. Microorganisms can produce abundant enzymes that can promote the metabolism to produce a variety of flavor substances. For example, glucosidase can directly degrade starch into glucose, and the glucose produced by decomposition enters the glycolysis pathway and citric acid cycle, etc., through which a variety of flavor substances and flavor precursor substances can be generated, providing a material basis for the formation of flavor of sauce-flavor *baijiu*. Researchers found that the primary fungi in the fermented grain of sauce-flavor *baijiu* produced in the Guizhou region include *Pichia*, *Saccharomyces*, *Thermoascus*, and *Wallemia*, while those in the fermented grain of sauce-flavor *baijiu* produced in the Beijing region are *Issatchenkia*, *Cryptococcus*, *Thermoascus,* and *Thermoyces* [9,13,19]. The microorganisms in the fermented grains are deeply involved in the saccharification of raw materials, the degradation of harmful substances, ethanol production, and the formation of flavor substances [5,20]. Clarifying the differences in microbial community composition and their contribution to the characteristic flavor substances in the brewing process of sauce-flavor *baijiu* produced in different regions is the key to unraveling the formation process of flavor substances.

Although the Beijing and Guizhou regions are both production areas for sauce-flavor *baijiu*, they are geographically distant and have significant differences in their environmental conditions [16]. Our previous studies found that the microbial community compositions and flavor compounds in the fermented grains of the fifth, sixth, and seventh rounds during the brewing process of sauce-flavor *baijiu* in Beijing and Guizhou regions had significant differences, ones that were closely related to the differences in environmental microorganisms [13,16]. However, it remains unclear if there are differences in the microbial community composition and profile of flavor compounds during the third-round fermentation process in the Beijing area, being crucial for sauce-flavor *baijiu* products. The current study aimed to investigate the temporal profile of microbial community and flavor compounds in fermented grains of sauce-flavor *baijiu* produced in the Beijing region during the third round of fermentation and to compare it with sauce-flavor *baijiu* produced in the Guizhou region to provide fundamental information for comprehending the formation of regional characteristic flavors.

## 2. Materials and Methods

### 2.1. Samples Collection

Samples came from a distillery in Beijing, China, in April 2023. The third-round fermentation process consists of two stages: 5 days heap fermentation and 30 days pit fermentation. The fermented grains of heap fermentation were sampled at 1, 3, and 5 days from the upper and lower layers. To reduce sample heterogeneity, the samples at 1, 3, and 5 days were ground and mixed as D1, D3, and D5, respectively [21]. The nine samples in pit fermentation came from the upper, middle, and lower layers at 0, 7, 14, 21, and 30 days. The collected samples were ground and mixed as J0S, J0Z, J0X, J7S, J7Z, J7X, J14S, J14Z, J14X, J21S, J21Z, J21X, J30S, J30Z, and J30X (Table 1). Finally, 18 samples were stored at −80 °C for further study.

### 2.2. DNA Extraction and Amplicon Sequencing

The E.Z.N.A.^®^ soil DNA Kit (Omega Bio-tek, Norcross, GA, USA) was used to extract microbial community genomic DNA from the samples. In brief, bacterial microflora analysis was performed for 338F (5-ACTCCTACGGGAGGCAGCAG-3′) and 806R (5′-GGACTACHVGGGTWTCTAAT-3′) by priming. Primers ITS1 (5′-TCCGTAGGTGAACCTGCGG-3′) and ITS2 (5′-GCTGCGTTCTTCATCGATGC-3′) were used for fungal microflora analysis. The sequencing of PCR amplication was carried out on the Illumina MiSeq PE300 platform/Nova-Seq PE250 platform (Illumina, San Diego, CA, USA) with the protocols of Majorbio Bio-Pharm Technology Co. Ltd. (Shanghai, China). The taxonomic classifications of the 16S rRNA and 18S rRNA gene sequences were analyzed by the RDP Classifier against the corresponding database at a confidence level of 70% [22].

### 2.3. Volatile Compounds Analysis

Samples mixed with saturated NaCl solution and 4-octanol (5 mg/L) were put into a 15 mL vial and sonicated for 30 min. The volatile compounds of the sample were extracted using a 50/30 μm DVB/CAR/PDMS fiber (Supelco, Bellefonte, PA, USA) and analyzed using an HS-SPME-GC-MS (TSQ 8000 Evo, Trace MS/GC, Thermo Fisher Scientific, Waltham, MA, USA). The oven temperature of gas chromatography (GC) was maintained at 40 °C for 3 min and was increased to 100 °C and held for 5 min (rate of 2 °C/min); then, it was increased to 150 °C and held for 2 min (rate of 2 °C/min), and finally it was increased to 230 °C and maintained for 5 min (rate of 10 °C/min). The flow rate of the helium carrier gas was 1 mL/min. Mass spectrometry (MS) was generated with an electron impact of 70 eV ionization energy and a full scan range from 30 to 400 amu [13].

### 2.4. Fermentation Parameters Analysis

In order to explore the intrinsic reasons for changes in microbial communities and flavor compounds, changes in four fermentation parameters during the fermentation process were investigated, namely, moisture, acidity, starch, and reducing sugar. International standard procedures were used to determine the starch content of the fermented grains (ISO 5377) [23]. The starch and reducing sugar content in the sample was determined by the Lane and Eynon constant titer method [24]. The moisture content was determined by gravimetry. Acidity was measured by acid–base titration [25,26].

### 2.5. Data Analysis

The microbial community compositions were calculated according to the relative abundance of OTUs at the genus level. Principal coordinate analysis (PCoA) was used to evaluate the ecological distances of the samples based on unweighted UniFrac distances. The relationships between microbial community structure and fermentation parameters were analyzed by redundancy analysis (RDA). The Spearman correlations between the top twenty genera in relative abundance, fermentation parameters, and concentrations of flavor compounds were calculated using OriginPro2019 (OriginLab Corporation, Northampton, MA, USA) and visualized using a heatmap.

## 3. Results

### 3.1. Temporal Profile of Microbial Community Composition

**Figure 1:**
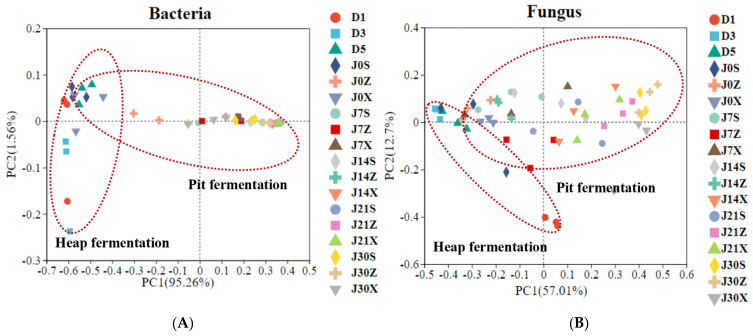
Beta diversity assessed by using an unweighted UniFrac principal coordinate analysis (PCoA) plot based on OTU level. (**A**) Bacterial communities; (**B**) fungal communities.

The temporal profile of microbial community composition in fermented grains was investigated by high-throughput sequencing. A total of 6,516,245 high-quality bacterial sequences and 6,727,486 high-quality fungal sequences were obtained from the samples, with 328 bacterial OTUs and 508 fungal OTUs. The rarefaction curves based on OTUs identified from samples gradually flattened as the number of samples increased, indicating that the sequencing depth was sufficient and most phylotypes were captured from a sufficient amount of data (Figures S1 and S2). The Shannon and Chao1 indexes of the bacterial community, which indicated the diversity and richness of the bacterial community, gradually increased during the heap fermentation process, reaching the highest levels at the end of heap fermentation, and then they gradually decreased with the extension of pit fermentation. The results showed that during the third round of fermentation, the diversity and richness of the bacterial community showed a trend of firstly an increase and then a decrease (Table S1). The Shannon index of the fungal community gradually decreased during heap fermentation and increased during the pit fermentation, ultimately reaching its highest level. The Chao1 index of the fungal community decreased during heap fermentation and fluctuated between 50.17 and 80.50 during pit fermentation, ultimately reaching the lowest level. These results indicated that the diversity of the fungal community increased while the richness decreased during the third round of the fermentation process (Table S2). The results of the principal coordinate analysis based on the profile of the microbial community showed that the bacterial and fungal community structure during heap fermentation was significantly different from that during pit fermentation (Figure 1).

**Figure 2:**
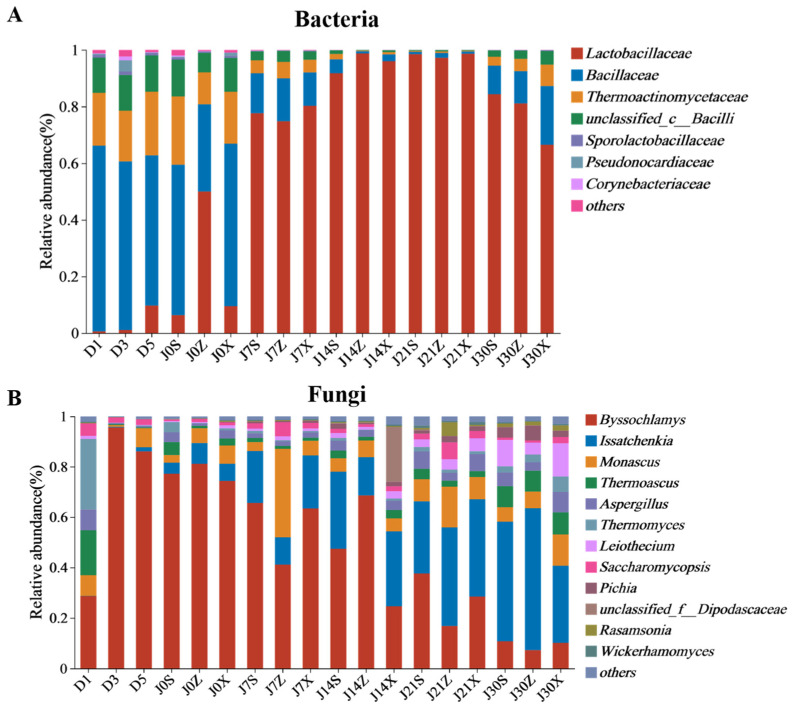
Temporal profile of microbial community structure in fermented grains during the third round of the fermentation process. (**A**) Bacterial; (**B**) fungal.

**Figure 3:**
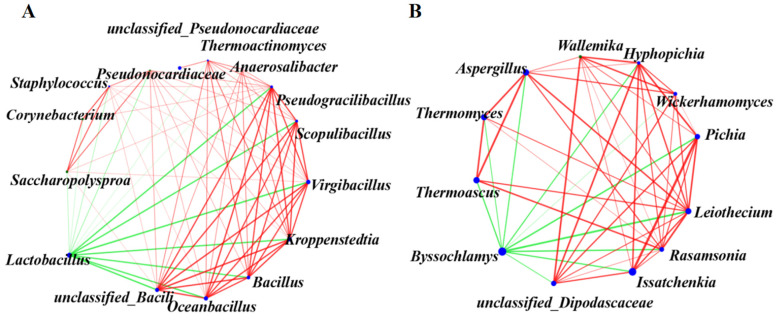
Correlation network diagram of microorganisms in fermented grains. (**A**) Bacterial correlation network diagram; (**B**) fungi correlation network diagram. The red line represents a positive correlation and the green line represents a negative correlation.

The SILVA rRNA and greengene databases were used for the classification and identification of the microorganisms [16]. In the third round of the fermentation process, 141 genera of bacteria were identified. There were 10 genera with relative abundances greater than 1%. The dominant bacteria genera included *Lactobacillus*, *Virgibacillus*, *Oceanobacillus*, *Kroppenstedtia*, *unclassified_c_Bacillus,* and *Bacillus* (Figure 2A). With the extension of time, the relative abundance of *Lactobacillus* showed an increasing trend, reaching a maximum of 98.77% at 21 days of pit fermentation. In contrast, the relative abundance of *Virgibacillus*, *Oceanobacillus*, *Kroppenstedtia,* and *unclassified_c_Bacillus* gradually decreased with time. By using Spearman’s correlation coefficient, the correlations between the top bacterial genera in terms of relative abundance were analyzed (Figure 3A). *Lactobacillus*, as the genus with the largest average relative abundance, was negatively correlated with all the other genera. *Virgibacillus*, *Oceanobacillus*, *Kroppenstedtia*, and *Bacillus* were positive correlated not only with each other but also with several genera (Figure 3A).

For fungi, a total of 209 genera were found in the samples, of which, *Byssochlamys* and *Issatchenkia* were the predominant genera, and *Monascus*, *Thermoascus*, *Aspergillus*, *Thermomyces*, *Leiothecium*, *Saccharomycopsis*, and *Pichia* were the subdominant genera (Figure 2B). The relative abundance of *Byssochlamys* reached a maximum of 95.16% at 3 days of heap fermentation and gradually decreased with time, reaching a minimum of 7.16% at the end of the pit fermentation. In contrast, the relative abundance of *Issatchenkia* showed an increasing trend with the increase in fermentation time, reaching a maximum at the end of the pit fermentation. Correlation analyses showed that *Byssochlamys* was negatively correlated with *Issatchenkia*, *Thermoascus*, *Aspergillus*, *Thermomyces*, *Leiothecium*, *Pichia*, *Hyphopichia*, *Rasamsonia*, *Wickerhamomyces,* and *unclassified_Dipodascaceae*. whereas *Issatchenkia* was positively correlated with *Pichia*, *Hyphopichia,* and *Rasamsonia* (Figure 3B).

### 3.2. Temporal Profile of Volatile Compounds

The temporal profile of volatile compounds in fermented grains was investigated by GC-MS. In total, 81 volatile compounds were detected in samples, including 14 alcohols, 32 esters, 7 acids, 7 aldehydes and ketones, 8 phenols, and 13 other flavor compounds. During the heap fermentation process, the concentrations of most volatile compounds increased gradually with time. Among them, ethyl palmitate, phenethyl alcohol, and ethanol, as relatively abundant esters and alcohols, reached 17.34 μg/g, 16.69 μg/g, and 10.63 μg/g at 5 days of heap fermentation, respectively (Table S3).

Compared to the fermented grain collected from heap fermentation, the fermented grain from pit fermentation had more diverse and abundant volatile compounds (Table S3). The concentrations of alcohols showed a fluctuating upward trend during pit fermentation, reaching a maximum concentration of 44.65 μg/g. Phenylethanol and ethanol were the most abundant alcohols, reaching maximum concentrations of 22.22 μg/g and 20.41 μg/g at 14 days of pit fermentation, respectively. In particular, the concentrations of ethanol in fermented grains located in the lower layer were slightly greater than those in the fermented grains located in the upper and middle layers. Five new alcohols, namely, 1-nonanol, benzyl alcohol, 3-methyl-1-butanol, (R)-(-)-1,2-propanediol, and (S,S)-2,3-butanediol, were detected during the pit fermentation. The concentration of esters in the sample increased with fermentation time, but the concentrations of esters were higher in the upper and middle layers than in the lower layer. Six esters were newly detected in the fermented grains of pit fermentation, namely, ethyl acetate, formic acid, hexylester, isoamyl acetate, ethyl 3-phenylpropionate, ethyl L(-)-lactate, and phenyl 4-hydroxyphosphate. More abundant acids were detected in the fermented grains from the pit fermentation than fermented grains from the heap fermentation. Among them, acetic acid, as the precursor of ethyl acetate, among others, increased rapidly from 0.20 μg/g to 0.90 μg/g after 14 days of pit fermentation.

### 3.3. Changes in Fermentation Parameters

**Figure 4:**
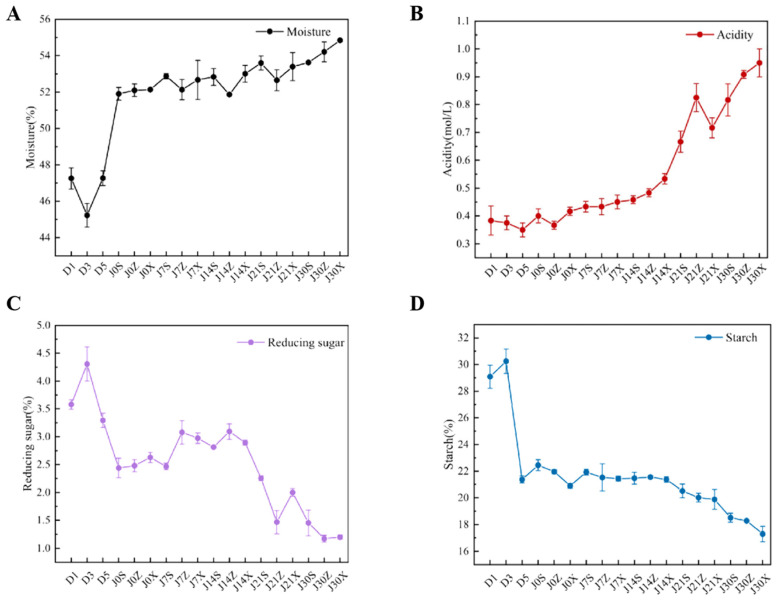
Changes in fermentation parameters during the third round of heap and pit fermentations. (**A**) Moisture; (**B**) acidity; (**C**) reducing sugar; (**D**) starch.

To study the causes of microbial community succession and changes in volatile compounds, the changes in moisture, starch, acidity, and reducing sugar during heap and pit fermentations were analyzed (Figure 4). The moisture of fermented grains during heap fermentation fluctuated between 47.26% and 51.50%, while it increased from 51.9% to 54.85% during pit fermentation. There was no significant difference in moisture content between the upper, middle, and lower layers at the beginning of pit fermentation, but the moisture content of the middle and lower layers was significantly higher than that of the upper layer at the end of pit fermentation (Table S4). With the extension of fermentation time, the acidity of fermentation particles increased gradually and reached the maximum value of 0.89 mol/L at the end of pit fermentation. In particular, the acidity of fermented grains in the upper layer was higher than those in the middle and lower layers at 30 days of pit fermentation (Table S4). The starch content showed an opposite trend to acidity, with the starch content gradually decreasing during the heap and pit fermentations, reaching 17.29% at 30 days of pit fermentation. Moreover, the starch content of fermented grains in the lower layer was lower than that in the upper and middle layers at the end of pit fermentation (Table S4). The reducing sugar content showed a decreasing trend with time. No significant difference in the reducing sugar content of fermented grains located in the upper, middle, and lower layers during pit fermentation was observed.

### 3.4. Correlations between Microbial Community Structure and Fermentation Parameters

**Figure 5:**
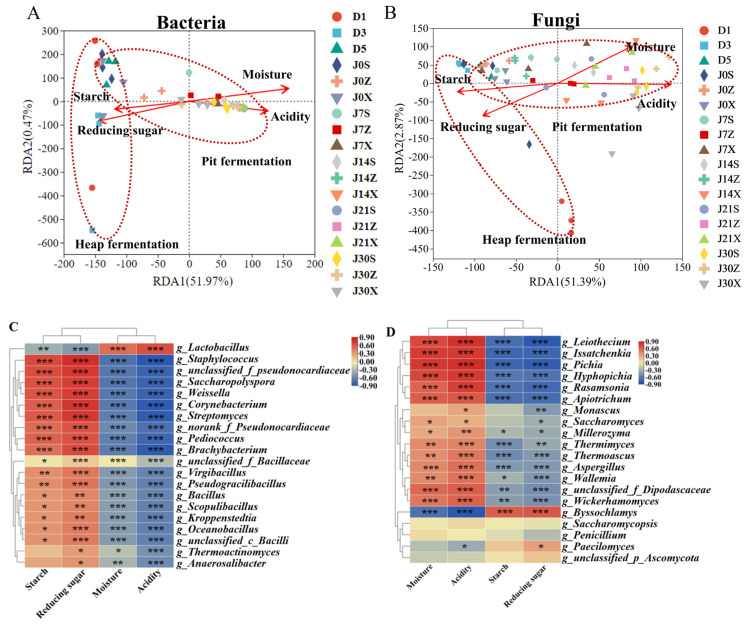
Correlation between fermentation parameters and microbial community. Redundancy analysis (RDA) of the dominant genera and the physicochemical indexes. (**A**) Bacterial; (**B**) fungal. Correlation heatmap between fermentation parameters and dominant genera. (**C**) Bacterial; (**D**) fungal. Asterisk shows significant correlations. *: *p* < 0.05, **: *p* < 0.01, ***: *p* < 0.001.

The RDA and Pearson’s correlation analyses were conducted to explore the causes of microbial community succession (Figure 5). The bacterial and fungal community succession during heap fermentation and the beginning of pit fermentation were positively correlated with the changes of starch and reducing sugar contents, and they were negatively correlated with the changes of moisture and acidity. As the pit fermentation time increased, the relationship between bacterial and fungal community structure and fermentation parameters changed. Between 7 and 30 days of pit fermentation, bacterial and fungal community succession were negatively correlated with changes of starch and reducing sugar contents and positively correlated with moisture and acidity. Pearson’s correlation analysis based on the top 20 microbial genera in relative abundance and fermentation parameters were conducted. The relative abundance of *Saccharopolyspora*, *Staphylococcus*, *Weissella*, and other dominant bacterial genera were positively correlated with starch and reducing sugar contents (*p* ≤ 0.001) and negatively correlated with moisture and acidity (*p* ≤ 0.001). In contrast, the relative abundance of *Lactobacillus* was negatively correlated with starch and reducing sugar contents (*p* ≤ 0.001) and positively correlated with moisture and acidity (*p* ≤ 0.001) (Figure 5C). The relative abundance of *Byssochlamys* was negatively correlated with moisture and acidity (*p* ≤ 0.001) and positively correlated with starch and reducing sugar contents (*p* ≤ 0.001). On the contrary, the relative abundance of *Hyphopichia*, *Apiotrichum*, *Rasamsonia*, *Issatchenkia*, *Pichia,* and *Leiothecium* was positively correlated with moisture and acidity (*p* ≤ 0.001) and negatively correlated with starch and reducing sugar contents (*p* ≤ 0.001) (Figure 5D).

### 3.5. Correlations between Microbial Community Structure and Volatile Compounds

**Figure 6:**
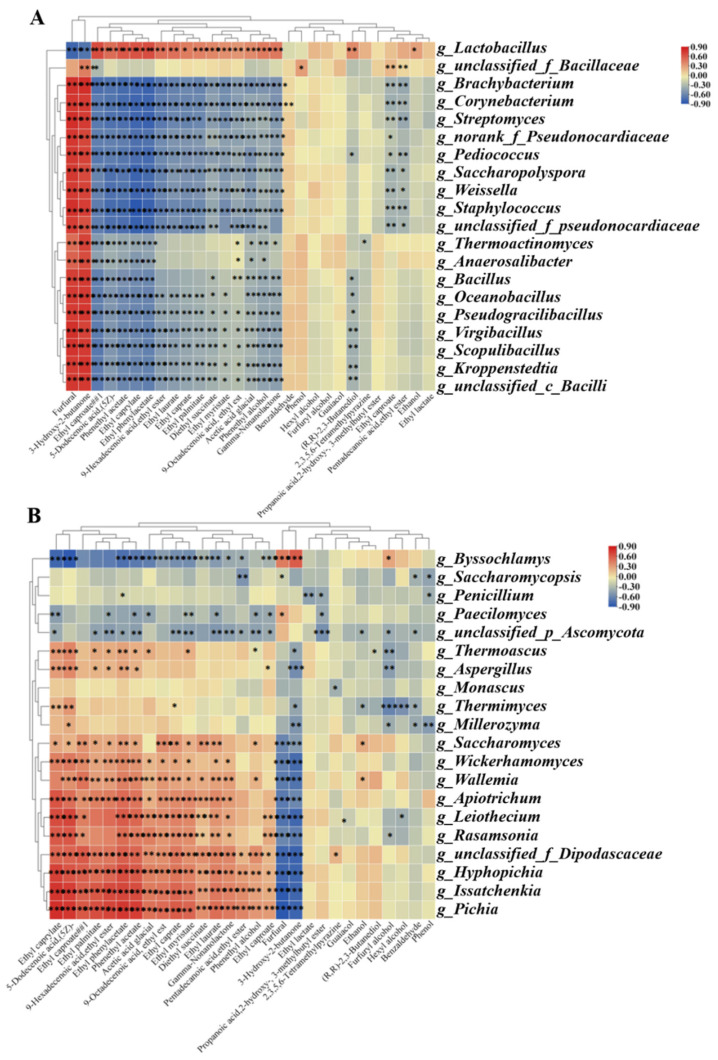
Relationship between the relative abundance of the key genera and concentrations of flavor compounds during third-round fermentation. (**A**) Correlation analysis between key bacterial genera and flavor compounds. (**B**) Correlation analysis between key fungal genera and flavor compounds. Red represents a positive correlation and blue represents a negative correlation. “*”, 0.01 < *p* < 0.05; “**”, 0.001 < *p* < 0.01; “***”, *p* < 0.001.

The relationship between the top 20 microbial genera in terms of relative abundance and the top 29 volatile compounds in terms of concentration was investigated to explore the causes of changes in the profile of flavor compounds in the third-round fermented grains (Figure 6). For bacterial genera, the relative abundance of *Lactobacillus* was positively correlated with ethyl acetate, ethyl caprylate, ethyl phenylacetate, phenethyl acetate, ethyl tetradecanoate, ethyl caprate, ethyl laurate, acetic acid, 5-dodecenoic acid, and (5Z)-, while it was negatively correlated with furfural and 3-hydroxy-2-butanone (*p* ≤ 0.001). The relative abundance of *Oceanobacillus*, *Pseudogracilibacillus*, *Virgibacillus*, *Scopulibacillus*, *Kroppenstedtia*, *unclassified_f_Bacillaceae*, *Brachybacterium*, *Corynebacterium*, *Streptomyces*, *norank_f_Pseudonocardiaceae*, *Pediococcus*, *Saccharopolyspora,* and *Weissella* was positively correlated with furfural and 3-hydroxy-2-butanone, while it was negatively correlated with ethyl caproate, phenethyl acetate, ethyl caprylate, ethyl phenylacetate, 9-hexadecenoic acid, ethyl ester, and phenethyl alcohol (*p* ≤ 0.001). For fungal genera, the relative abundance of *Byssochlamys* was positively correlated with furfural and 3-hydroxy-2-butanone, while it was negatively correlated with 5-dodecenoicacid, (5Z)-, ethyl caprylate, phenethyl acetate, ethyl phenylacetate, ethyl caprate, ethyl myristate, 9-octadecenoic acid, ethyl est, acetic acid glacial, and ethyl caproate. The relative abundance of *Issatchenkia*, *Picha,* and *Hyphopichia* was negatively correlated with furfural and 3-hydroxy-2-butanone but was positively correlated with ethyl caprylate, ethyl caproate, ethyl palmitate, 9-hexadecenoic acid, ethyl ester, ethyl phenylacetate, phenethyl acetate, acetic acid glacial, 9-Octadecenoic acid, ethyl est, ethyl caprylate, gamma-nonanolactone, and phenethyl alcohol. In addition, the relative abundance of *Wickerhamomyces*, *Wallemia*, *Apiotrichum,* and *Rasamsonia* was positively correlated with ethyl caprylate and ethyl phenylacetate (Figure 6).

## 4. Discussion

The flavor of sauce-flavor *baijiu* produced in Beijing and Guizhou is different, but the reason is unclear. The current study investigated the temporal profile of the microbial community and flavor compounds in the fermented grains of sauce-flavor *baijiu* produced in the Beijing region during the third round of fermentation; among them, the dominant fungi included *Byssochlamys*, *Issatchenkia*, *Monascus*, *Thermoascus*, *Aspergillus,* and *Thermomyces*, while the dominant bacteria included *Lactobacillus*, *Virgibacillus*, *Oceanobacillus*, *Kroppenstedtia,* and *Bacillus* (Figure 2). In contrast, the dominant fungi in third-round fermented grains of sauce-flavor *baijiu* in the Guizhou region included *Pichia, Zygosaccharomyces, Monascus*, *Saccharomyces, Thermoascus*, *Aspergillus*, and *Thermomyces*, while the dominant bacteria included *Lactobacillus*, *Virgibacillus*, *Oceanobacillus*, *Kroppenstedtia*, *Sphingobacterium*, *Enterobacter*, *Pantoea*, and *Bacillus* [27,28]. This study was the first to compare and analyze the microbial community composition in the fermented grains of sauce-flavor *baijiu* in the Beijing and Guizhou regions during the third round of fermentation. The results showed that the difference in fungi in the fermented grains of sauce-flavor *baijiu* between the Beijing area and Guizhou area was more significant than that of the bacteria.

To better understand the differences in the microbial community composition of sauce-flavor *baijiu* in different geographic areas, we compared the microbial community structure in fermented grains from the third, fifth, and sixth rounds of fermentation in the Beijing region. We found that the dominant bacterial genera were approximately the same among the three rounds, with *Lactobacillus*, *Virgibacillus*, and *Oceanobacillus* as the dominant bacterial genera. However, there were slight differences in the composition of the fungal genera, with the dominant fungal genera in the third round being *Byssochlamys*, *Issatchenkia,* and *Monascus*; the dominant fungal genera in the fifth round being *Issatchenkia*, *Thermoascus*, and *Thermomyces*; and the dominant fungal genera in the sixth round being *Issatchenkia*, *Thermoascus,* and *Byssochlamys* [16]. Our previous study found that differences in the abundance of *Pichia* and *Issatchenkia* in the brewing environment partially contributed to the differences in microbial composition of the fermented grains in the Guizhou and Beijing regions [16]. The differences in the microbial community structure of fermented grains from different rounds in the Beijing region were smaller than those from the Beijing and Guizhou regions, suggesting that the sauce-flavor *baijiu* microbiome is geography dependent [15].

There is an inevitable relationship between the flavor of sauce-flavor *baijiu* and its microorganisms [15,29]. The volatile compounds analysis showed that esters, such as ethyl caproate, ethyl acetate, ethyl lactate, ethyl caprylate, ethyl phenyl acetate, phenethyl acetate, ethyl myristate, and ethyl palmitate, and alcohols, such as ethanol and phenylethanol, were abundant in the fermented grains (Table S3). In contrast, the main flavor compounds in the fermented grains of the Guizhou region included ethyl lactate, ethyl phenylacetate, ethyl propionate, 2-pentyl-furan, 3-hydroxy-2-butanone, 4-ethyl-2-methoxyphenol, and (2r,3r)-butanediol [15]. Moreover, butanoic acid, 3-methylbutanoic acid, tetramethylpyrazine, and other substances were considered to be the chemical markers and flavor-related chemicals in the fermented grains of sauce-flavor *baijiu* in the Guizhou region; however, only a relatively low concentration of tetramethylpyrazine was detected in samples from the Beijing region [15] (Table S3), suggesting that the profile of flavor compounds in the fermented grains of sauce-flavor *baijiu* in the Beijing and Guizhou regions had significant differences. The plentiful flavor compounds in the fermented grains of the fifth and sixth rounds in the Beijing region were mainly esters, such as ethyl caproate, ethyl acetate, ethyl lactate, ethyl caprylate, and ethyl phenyl acetate, which was similar to that of the third round [13] (Table S3). High concentrations of 1-butanol, 3-methyl-, acetic acid, butyric acid, capric acid, and ammonium acetate were detected in the fermented grains of the fifth and sixth rounds, but there were low concentrations of these flavor compounds or they were not detected in the fermented grains of the third round [13] (Table S3). These results showed that differences in the profile of flavor compounds in fermented grains from different rounds in the Beijing region were smaller than those in the fermented grains from the Beijing and Guizhou regions.

One of the reasons for the differences in the profile of flavor compounds in the fermented grains of sauce-flavor *baijiu* from the Beijing and Guizhou regions is the difference in microbial community composition. Tan et al. reported that the interactions among *Pichia*, *Zygosaccharomyces*, and *Lactobacillus* significantly contributed to the formation of 2-acetylpyrrole, tetramethylpyrazine, β-ethylphenethyl alcohol, and benzoic acid during the fermentation process of sauce-flavor *baijiu* from Guizhou region [15]. In contrast, the concentrations of ethyl acetate, ethyl caprylate, and ethyl phenylacetate, as well as the concentrations of acetic acid and cis-5-dodecenoic acid, were positively correlated with the relative abundance of *Lactobacillus*, *Issatchenkia,* and *Pichia* during the fermentation process of sauce-flavor *baijiu* from the Beijing region (Figure 6). Another reason for differences in the profile of flavor compounds is the differences in the fermentation parameters of fermented grains. The generation of tetramethylpyrazine during the brewing process of sauce-flavor *baijiu* mainly involves two methods: the Maillard reaction and microbial synthesis [29]. The type and concentration of the Maillard reaction substrate in fermented grains have a remarkable effect on the content of tetramethylpyrazine [29]. Dynamic changes in microbial community composition cause changes in the fermentation parameters of fermented grains such as temperature, acidity, and glucose content (Figure 4), which in turn affect the synthesis of flavor compounds such as tetramethylpyrazine [29]. The acidity during the third-round fermentation in Beijing was significantly lower than that in Guizhou, so the concentration of tetramethylpyrazine detected in the fermented grains of sauce-flavor baijiu in the Beijing region was found to be lower than that in the Guizhou region [15]. Therefore, a comprehensive understanding of microbial community succession and fermentation parameter changes during the fermentation process is essential for analyzing the formation of characteristic flavor substances.

The microbial community succession in the third round of fermentation of sauce-flavor *baijiu* in the Beijing region was investigated. With the extension of fermentation time, the relative abundance of *Lactobacillus* increased, while the relative abundance of *Virgibacillus*, *Oceanobacillus,* and *Kroppenstedtia* gradually decreased (Figure 2A). The relative abundance of *Byssochlamys* reached a maximum of 95.16% at the 3 days of heap fermentation and then gradually decreased with time, while *Issatchenkia* gradually increased, becoming the genus with the highest relative abundance at the end of pit fermentation (Figure 2B). The pattern of microbial community succession during the third round of fermentation was similar to that during the fifth and sixth rounds of fermentation of sauce-flavor *baijiu* in the Beijing region [13].

The drivers of microbial community succession in fermented grains include microbial interactions and changes in fermentation parameters. *Lactobacillus*, as the dominant bacterial genus in the fermented grains of sauce-flavor *baijiu*, can inhibit the growth of acid-intolerant microorganisms by increasing the acidity of fermented grains through the production of lactic acid and acetic acid, as well as being able to antagonize other Gram-negative bacteria through the production of bacteriocins [21,30,31]. It was observed that the acidity of fermented grains increased as the relative abundance of *Lactobacillus* was boosted, which positively drove the bacterial community succession (Figure 2A, Figure 4 and Figure 5A). Moreover, correlation analysis showed that *Lactobacillus* was negatively correlated with *Virgibacillus*, *Oceanobacillus,* and *Kroppenstedtia* (Figure 3). The acidity of fermented grains also positively drove the fungal community succession (Figure 5B). It was observed that the relative abundance of *Issatchenkia* and *Pichia* increased with the increased acidity of fermented grains (Figure 2B and Figure 4). *Pichia*, the dominant non-Saccharomyces fungal genus in the fermented grains of the Guizhou region, not only decomposes lactic acid to maintain vigorous growth under acid stress but also inhibits the growth of filamentous fungi and *Bacillus* by producing toxins and organic acids [28,29,32,33,34]. *Issatchenkia* can thrive in varied fermentation environments, owing to its high acid and temperature tolerance, while producing various acids and ethanol to shape the microbial community structure [35,36]. The acidity of fermented grains was positively correlated with the relative abundance of *Issatchenkia* and *Pichia* (Figure 5D), indicating that acidity had a remarkable driving effect on the microbial community succession during the third round of fermentation of sauce-flavor *baijiu* in the Beijing region.

**Figure 7:**
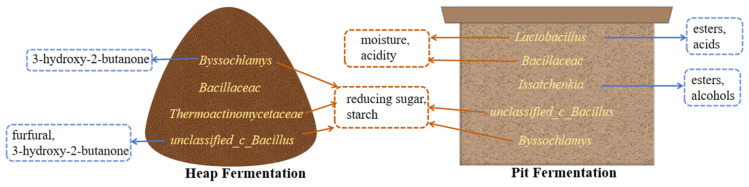
A summary map of dominant microorganisms and potential functions.

In summary, profiles of heap fermentation and pit fermentation were drawn based on the obtained results from this work to directly reflect the relationship between dominant microorganisms, fermentation parameters, and volatile compounds in the *baijiu* fermentation process (Figure 7). The fermentation process of sauce-flavor *baijiu* takes a year, during which it needs to go through seven rounds of *baijiu* extraction. The quality of the third round of *baijiu* is better, and the amount is large in the blending process of the production of sauce-flavor *baijiu*, playing a vital role in the flavor and quality of the product. Our research provides an extremely important scientific basis for understanding the formation of the special flavor of sauce-flavor *baijiu* in different regions.

## 5. Conclusions

The temporal profile of the microbial community and volatile compounds during the third round of fermentation of sauce-flavor *baijiu* in the Beijing region were studied. The results showed that *Lactobacillus*, *Virgibacillus*, *Oceanobacillus*, *Kroppenstedtia*, *unclassified_c_Bacillus*, *Bacillus,* and *unclassified_f_Bacillaceae* were the core bacterial flora, and *Byssochlamys*, *Issatchenkia*, *Monascus*, *Thermoascus*, *Aspergillus*, *Thermomyces*, *Leiothecium*, *Saccharomycopsis*, and *Pichia* were the core fungal flora. The acidity of fermented grains drove the microbial community succession. A total of 81 volatile compounds were identified in the 18 samples, among which esters and alcohols were abundant, and their concentrations increased with time. The differences in the profile of flavor compounds and microbial community structure in fermented grains from different rounds in the Beijing region were smaller than those in the fermented grains from the Beijing and Guizhou regions. The difference in microbial community composition and their resulted differences in fermentation parameters of fermented grains were found to be responsible for the differences in the profiles of flavor compounds between sauce-flavor *baijiu* produced in the Beijing and Guizhou regions.

## Figures and Tables

**Table 1:** Summary of all the samples collected.

Sample Name	Sampling Location	Sampling Time
D1	heap fermentation mixing the upper and lower layers	1 days
D3	heap fermentation mixing the upper and lower layers	3 days
D5	heap fermentation mixing the upper and lower layers	5 days
J0S	pit fermentation upper layers	0 days
J0Z	pit fermentation middle layers	0 days
J0X	pit fermentation lower layers	0 days
J7S	pit fermentation upper layers	7 days
J7Z	pit fermentation middle layers	7 days
J7X	pit fermentation lower layers	7 days
J14S	pit fermentation upper layers	14 days
J14Z	pit fermentation middle layers	14 days
J14X	pit fermentation lower layers	14 days
J21S	pit fermentation upper layers	21 days
J21Z	pit fermentation middle layers	21 days
J21X	pit fermentation lower layers	21 days
J30S	pit fermentation upper layers	30 days

## Data Availability

The original contributions presented in the study are included in the article/Supplementary Materials. Further inquiries can be directed to the corresponding author.

## Supplementary Materials

The following supporting information can be downloaded at https://www.mdpi.com/article/10.3390/foods13050670/s1. Figure S1. Rarefaction curves of bacterial Chao1 index (A) and Shannon index (B). Figure S2. Rarefaction curves of fungal Chao1 index (A) and Shannon index (B). Figure S3. The seven rounds of the manufacturing process of the sauce-flavor *baijiu*. Table S1. Shannon and Chao1 indexes for the bacterial community. Groups with different letters mean significant differences (*p* < 0.05) as determined by one-way ANOVA Tukey’s post hoc test. Table S2. Shannon and Chao 1 indexes for fungal community. Groups with different letters mean significant differences (*p* < 0.05) as determined by one-way ANOVA Tukey’s post hoc test. Table S3. Temporal profile of volatile compounds in fermented grains of the third round of fermentation (μg/g). Table S4. Changes of fermentation parameters in the upper, middle, and lower layers of fermented grains. Groups with different letters mean significant differences (*p* < 0.05) as determined by one-way ANOVA Tukey’s post hoc test.
