# Supplementary material for: Temporal Profile of the Microbial Community and Volatile Compounds in the Third-Round Fermentation of Sauce-Flavor baijiu in the Beijing Region
Source: Foods. 2024 Feb 22;13(5):670. doi: 10.3390/foods13050670 (PMC10931104; doi:10.3390/foods13050670)
Supplement: Supplementary file 1 [file foods-13-00670-s001.zip › foods-2865364-supplementary.pdf]

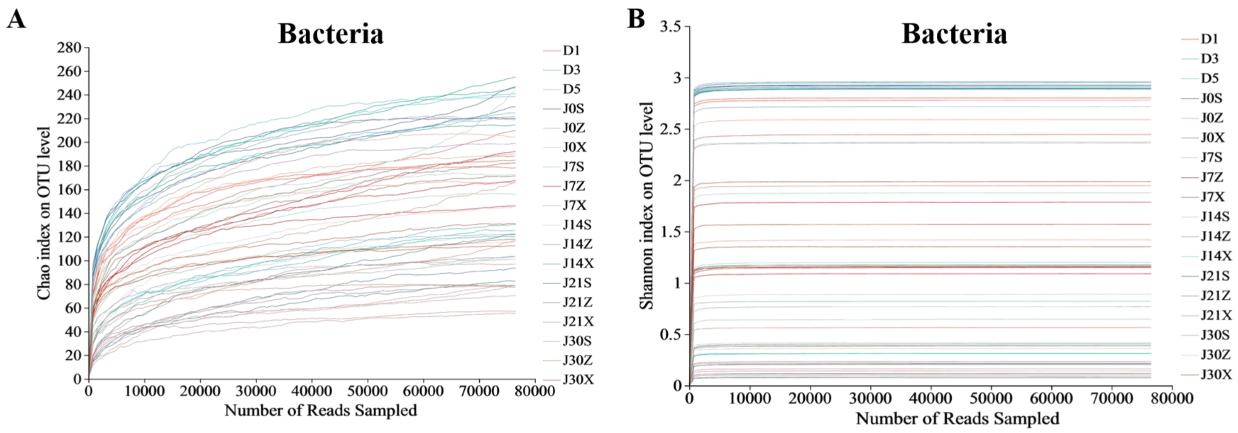

Supplementary Fig.S1 Rarefaction curves of bacterial Chao 1 index (A) and Shannon index (B)

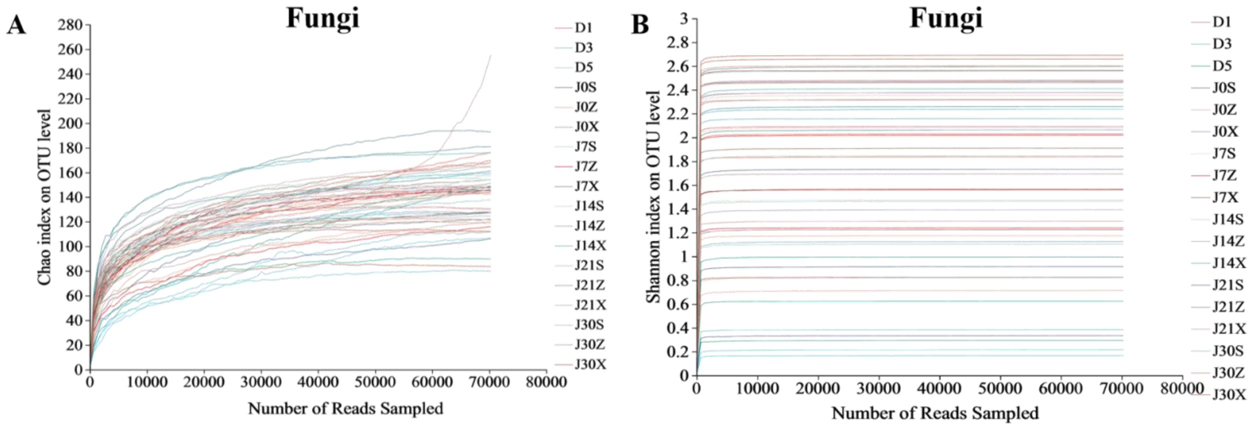

Supplementary Fig.S2 Rarefaction curves of fungal Chao 1 index (A) and Shannon index (B)

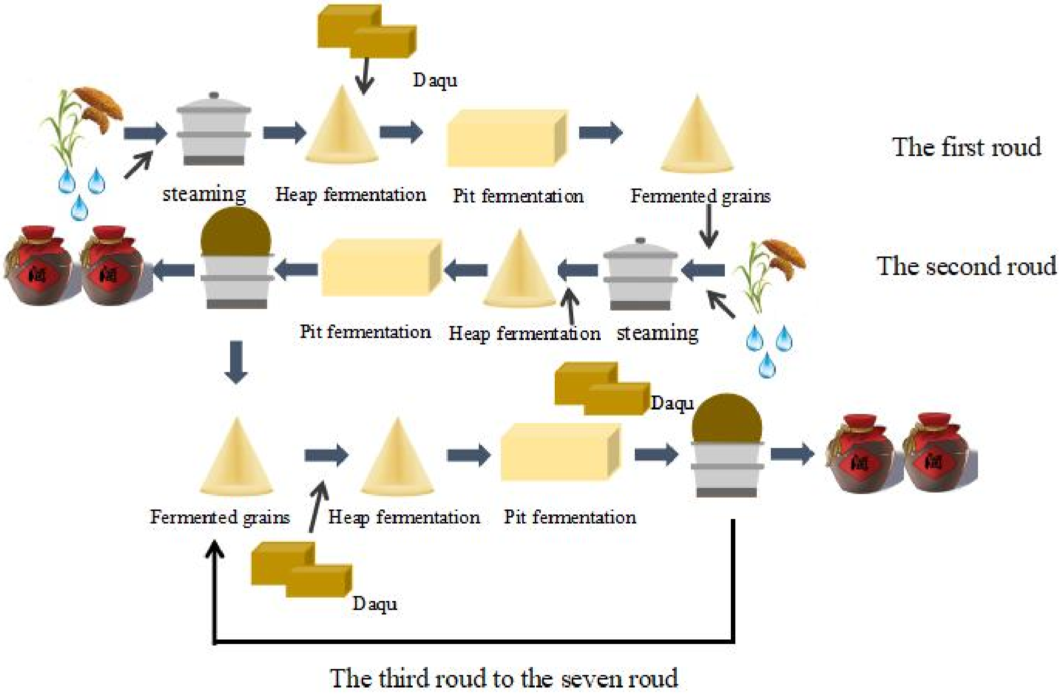

Supplementary Fig. S3. The seven rounds of manufacturing process of sauce-flavor *baijiu*.

Supplementary Table S1. Shannon and Chao 1 indexes for bacterial community. Groups with different letters mean significant differences (*P*<0.05) as determined by one-way ANOVA Tukey's post hoc test.

Sample	Shannon	Chao 1
D1	1.63 ± 0.09^{ab}	72.27 ± 0.90^{abcd}
D3	1.86 ± 0.21^{a}	97.56 ± 4.74^{a}
D5	1.88 ± 0.02^{a}	94.23 ± 4.97^{ab}
J0S	1.89 ± 0.03^{a}	72.74 ± 3.44^{bcde}
J0Z	1.52 ± 0.33^{ab}	69.35 ± 11.54^{cde}
J0X	1.86 ± 0.11^{a}	75.05 ± 6.72^{bcd}
J7S	0.90±0.35cd	59.88 ± 5.08^{cdef}
J7Z	1.00 ± 0.22^{cd}	94.30 ± 11.30^{abc}
J7X	0.87 ± 0.01^{cd}	52.08 ± 10.34^{efg}
J14S	0.30 ± 0.02^{ef}	44.82 ± 4.68^{fgh}
J14Z	0.05 ± 0.00^{f}	24.00 ± 5.00^{h}
J14X	0.25 ± 0.01^{f}	37.25 ± 0.25^{gh}
J21S	0.10 ± 0.04^{f}	32.50 ± 6.50^{h}
J21Z	0.18 ± 0.06^{f}	21.11 ± 1.64^{h}
J21X	0.10 ± 0.02^{f}	28.00 ± 4.97^{h}
J30S	0.71 ± 0.13^{de}	30.73 ± 3.80^{gh}
J30Z	0.37 ± 0.07^{de}	41.00 ± 7.00^{gh}
J30X	1.22 ± 0.17^{bc}	44.50 ± 1.50^{def}

Supplementary Table S2. Shannon and Chao 1 indexes for fungal community. Groups with different letters mean significant differences (*P*<0.05) as determined by one-way ANOVA Tukey's post hoc test.

Sample	Shannon	Chao 1
D1	$1.78{\pm}0.05^{ab}$	$70.43{\pm}7.57^{ab}$
D3	$0.24{\pm}0.09^{f}$	$54.83{\pm}8.83^{abc}$
D5	$0.75{\pm}0.18^{e}$	$50.13{\pm}3.72^{c}$
J0S	$0.86{\pm}0.50^{e}$	$61.82{\pm}0.32^{abc}$
J0Z	$0.77{\pm}0.13^{e}$	$61.71{\pm}3.29^{abc}$
J0X	$1.10{\pm}0.09^{de}$	$65.64{\pm}4.96^{abc}$
J7S	$1.17{\pm}0.19^{bcd}$	$66.76{\pm}6.12^{ab}$
J7Z	$1.41{\pm}0.25^{abc}$	$54.47{\pm}8.75^{abc}$
J7X	$1.13{\pm}0.27^{de}$	$61.53{\pm}5.70^{abc}$
J14S	$1.51{\pm}0.25^{abc}$	$60.50{\pm}5.50^{abc}$
J14Z	$1.17{\pm}0.20^{bcd}$	$57.37{\pm}0.45^{abc}$
J14X	$1.58{\pm}0.32^{abc}$	$59.63{\pm}6.08^{abc}$
J21S	$1.83{\pm}0.18^{ab}$	$80.50{\pm}8.00^{a}$
J21Z	$1.89{\pm}0.06^{ab}$	$54.98{\pm}2.77^{abc}$
J21X	$1.78{\pm}0.12^{ab}$	$64.47{\pm}2.08^{abc}$
J30S	$1.85{\pm}0.10^{ab}$	$56.33{\pm}2.49^{abc}$
J30Z	$1.66{\pm}0.21^{abc}$	$50.50{\pm}11.79^{c}$
J30X	$2.13{\pm}0.12^{a}$	$50.17{\pm}11.72^{c}$

Table S3. Changes of fermentation parameters in the upper, middle and lower layers of fermented grains. Groups with different letters mean significant differences (*P*<0.05) as determined by one-way ANOVA Tukey's post hoc test.

Sample	Mositure（%）	Acidity（mol/L）	Redecing sugar（%）	Starch（%）
J0S	51.90±0.35^{fg}	0.40±0.03^{fg}	2.44±0.17^{de}	22.45±0.41^{a}
J0Z	52.10±0.35^{efg}	0.37±0.01^{g}	2.48±0.11^{de}	21.96±0.15^{ab}
J0X	52.14±0.06^{efg}	0.42±0.01^{fg}	2.62±0.09^{cd}	20.90±0.17^{cd}
J7S	52.87±0.15^{cde}	0.43±0.02^{ef}	2.47±0.06^{de}	21.93±0.22^{ab}
J7Z	52.13±0.56^{efg}	0.43±0.03^{ef}	3.08±0.21^{a}	21.53±1.02^{bc}
J7X	52.67±1.07^{defg}	0.45±0.02^{ef}	2.97±0.09^{ab}	21.43±0.18^{bc}
J14S	52.83±0.46^{cdef}	0.46±0.01^{ef}	2.81±0.02^{bc}	21.47±0.45^{bc}
J14Z	51.87±0.06g	0.48±0.01^{de}	3.09±0.14^{a}	21.55±0.11^{bc}
J14X	53.00±0.46^{cdf}	0.53±0.02^{d}	2.89±0.05^{ab}	21.37±0.21^{bc}
J21S	53.60±0.38^{bc}	0.67±0.04^{c}	2.25±0.05^{e}	20.51±0.51^{de}
J21Z	52.65±0.58^{defg}	0.83±0.05^{b}	1.47±0.21^{g}	20.02±0.33^{e}
J21X	53.39±0.77^{bcd}	0.72±0.04^{c}	2.00±0.07^{f}	19.88±0.75^{e}
J30S	53.62±0.04^{bc}	0.82±0.06^{b}	1.45±0.23^{g}	18.51±0.34^{f}
J30Z	54.21±0.55^{ab}	0.91±0.01^{a}	1.17±0.06^{h}	18.28±0.08^{f}
J30X	54.85±0.06^{a}	0.95±0.05^{a}	1.20±0.04^{h}	17.29±0.57^{g}

Table S4. Changes of fermentation parameters in the upper layer of fermented grains, middle layer of fermented grains and lower layer of fermented grains. Groups with different letters mean significant differences (*P*<0.05) as determined by one-way ANOVA Tukey's post hoc test.

Sample	Mositure（%）	Acidity（mol/L）	Redecing sugar（%）	Starch（%）
J0S	51.90±0.35^{c}	0.40±0.03^{c}	2.44±0.17^{b}	22.45±0.41^{a}
J7S	52.87±0.15^{b}	0.43±0.02^{c}	2.47±0.06^{b}	21.93±0.22^{ab}
J14S	52.83±0.46^{b}	0.46±0.01^{c}	2.81±0.02^{a}	21.47±0.45^{b}
J21S	53.60±0.38^{a}	0.67±0.04^{b}	2.25±0.05^{b}	20.51±0.51^{c}
J30S	53.62±0.04^{a}	0.82±0.06^{a}	1.45±0.23^{c}	18.51±0.34^{d}
J0Z	52.10±0.35^{b}	0.37±0.01^{d}	2.48±0.11^{b}	21.96±0.15^{a}
J7Z	52.13±0.56^{b}	0.43±0.03^{c}	3.08±0.21^{a}	21.53±1.02^{a}
J14Z	51.87±0.06^{b}	0.48±0.01^{c}	3.09±0.14^{a}	21.55±0.11^{a}
J21Z	52.65±0.58^{b}	0.83±0.05^{b}	1.47±0.21^{c}	20.02±0.33^{b}
J30Z	54.21±0.55^{a}	0.91±0.01^{a}	1.17±0.06^{d}	18.28±0.08^{c}
J0X	52.14±0.06^{c}	0.42±0.01^{d}	2.62±0.09^{b}	20.90±0.17^{a}
J7X	52.67±1.07^{bc}	0.45±0.02^{d}	2.97±0.09^{a}	21.43±0.18^{a}
J14X	53.00±0.46^{bc}	0.53±0.02^{c}	2.89±0.05^{a}	21.37±0.21^{a}
J21X	53.39±0.77^{b}	0.72±0.04^{b}	2.00±0.07^{c}	19.88±0.75^{b}
J30X	54.85±0.06^{a}	0.95±0.05^{a}	1.20±0.04^{d}	17.29±0.57^{c}
